# Puncture mechanics of cnidarian cnidocysts: a natural actuator

**DOI:** 10.1186/1754-1611-3-17

**Published:** 2009-09-28

**Authors:** Shawn C Oppegard, Peter A Anderson, David T Eddington

**Affiliations:** 1 Department of Bioengineering, University of Illinois at Chicago, Chicago, IL 60607, USA; 2 Whitney Laboratory for Marine Bioscience and Department of Physiology and Functional Genomics, University of Florida, St. Augustine, FL 32080, USA; 3 Department of Biopharmaceutical Sciences, University of Illinois at Chicago, Chicago, IL 60612, USA

## Abstract

**Background:**

Cnidocysts isolated from cnidarian organisms are attractive as a drug-delivery platform due to their fast, efficient delivery of toxins. The cnidocyst could be utilized as the means to deliver therapeutics in a wearable drug-delivery patch. Cnidocysts have been previously shown to discharge upon stimulation via electrical, mechanical, and chemical pathways. Cnidocysts isolated from the Portuguese Man O' War jellyfish (*Physalia physalis*) are attractive for this purpose because they possess relatively long threads, are capable of puncturing through hard fish scales, and are stable for years.

**Results:**

As a first step in using cnidocysts as a functional component of a drug delivery system, the puncture mechanics of the thread were characterized. Tentacle-contained cnidocysts were used as a best-case scenario due to physical immobilization of the cnidocysts within the tentacle. *Ex vivo *tentacle-contained cnidocysts from *Physalia *possessed an elastic modulus puncture threshold of approximately 1-2 MPa, based on puncture tests of materials with a gamut of hardness. Also, a method for inducing discharge of isolated cnidocysts was found, utilizing water as the stimulant. Preliminary lectin-binding experiments were performed using fluorophore-conjugated lectins as a possible means to immobilize the isolated cnidocyst capsule, and prevent reorientation upon triggering. Lectins bound homogeneously to the surface of the capsule, suggesting the lectins could be used for cnidocyst immobilization but not orientation.

**Conclusion:**

Cnidocysts were found to puncture materials up to 1 MPa in hardness, can be discharged in a dry state using water as a stimulant, and bind homogeneously to lectins, a potential means of immobilization. The information gained from this preliminary work will aid in determining the materials and design of the patch that could be used for drug delivery.

## Background

**Figure 1:**
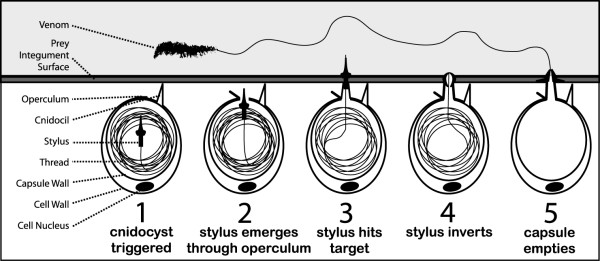
**Illustration depicting cnidocyst discharge into prey**. The cnidocil is a mechanosensory element on the apical surface of the cnidocyte. Upon appropriate stimulation, including mechanical stimulation of the cnidocil, the cnidocyst is triggered to rapidly evert its stinging thread into the potential predator or prey. Venom is then delivered into the contacting organism.

Cnidarians (including jellyfish, sea anemones, and corals) utilize the cnidocyst as a tool to capture prey and inject venom as shown in figure 1. Cnidocysts are produced by cnidocytes and are typically located in the tentacle of the organism where they serve the functions of prey immobilization, predator defense, and locomotion. Cnidocysts consist of a rigid capsule wall made of collagen-like proteins, enclosing a tightly coiled stinging thread that rapidly everts from the capsule upon discharge. The thread penetrates the prey's integument and introduces venom. This discharge event is one of the fastest processes in the animal kingdom, characterized by a theoretical thread tip pressure of nearly 7 GPa and a velocity of over 15 m/s [1].

Cnidocyst discharge is still poorly understood, owing to the nanosecond duration of the process. Cnidocyte discharge *in vivo *typically requires the near simultaneous application of chemical and mechanical stimuli [2-4]. The mechanosensory aspect of discharge is mediated by stimulation of the cnidocyte cnidocil, which is essentially a finger-like projection at the apical end of the cell that initiates an intracellular signaling cascade upon mechanical perturbation by a predator or prey [5]. The chemosensory component is less well-understood, but is thought to include both contact and distance chemoreceptors, and presumably is required to ensure the organism only discharges its cnidocytes when the probability of capturing food is high [2].

A few theories have been proposed to explain the rapid process of discharge once the cnidocyst is stimulated by the cell. Discharge was initially thought to be mediated by a high internal osmotic pressure, mechanical energy stored within the capsule wall, and a sudden increase in hydrostatic pressure [6]. High internal calcium concentrations have been measured intracellularly, and upon discharge, Ca^2+ ^ions are rapidly exocytosed as the cnidocyst membrane fuses with the cnidocyte membrane and the cyst comes into contact with sea water. Within the capsule, an anionic network of polyglutamic acid is bound and crosslinked by these calcium ions, maintaining a low osmotic gradient [7,8]. As a prelude to discharge, the calcium ions are thought to dissociate from the poly-glutamic peptides and exit the capsule. The poly-Glu peptides remain within the cyst, and the now uncrosslinked monomers of the anionic network increase the number of osmotically active species [6,9]. A rapid influx of sea water occurs, yielding a large, sudden increase in hydrostatic pressure, inducing the thread to discharge. The force of the hydrostatic pressure causes the thread to rapidly evert with a force sufficient to puncture through even the hard scales of some bony fish.

More recent research suggests that a proton gradient established across the cyst wall may explain the extremely quick initial exocytosis of the thread [10]. The protons unbind from polymer matrix and leave the capsule, leading to electrostatic repulsion of the polyglutamic acid chains. This repulsion ultimately results in an increase in pressure on the capsular wall and an increase in the capsule volume. The uptake of inorganic cations and water may change the pH and osmotic pressure within the capsule and aid in evagination of the thread due to a conformational change. However, the rapid speed cannot be explained by this influx of ions alone and thus is argued to be due to the fast translocation of protons.

Cnidocysts offer an attractive solution to transdermal drug delivery as they are essentially a natural microscale injection module that could be integrated into a patch-like device. The cnidocysts could be loaded with drug and upon discharge would evert their threads, injecting the patient with the drug. Specifically, the work outlined in this paper explores the use of cnidocysts isolated from the siphonophore *Physalia physalis *(Portuguese Man O' War) as a first step towards this goal. The use of this animal is attractive for a number of reasons. The isolated cnidocysts after removal from the tentacle cnidocytes are capable of being stored for more than one year in water at 4°C (to minimize bacterial contamination), while still able to discharge upon stimulation (unpublished observations). Also, the penetration of cnidocyst threads would be painless due to the microscale diameter of the thread. Pain experienced by a cnidarian sting is due to the injection of venom as opposed to the mechanical disturbance of the thread. For example, a 33 gauge needle is the smallest diameter needle commonly used for clinical application, possessing about a 90 μm tip diameter. In contrast, the cnidocyst thread tip diameter is about 2 μm [11,12]; nearly two orders of magnitude smaller. Finally, *Physalia *itself is capable of puncturing the exoskeleton of certain crustaceans and possesses relatively long threads (many over 1 mm in length). These attributes permit more flexibility in choosing the materials and design of the patch.

As a first step in using cnidocysts isolated from *Physalia *in a drug-delivery patch, the discovery of a simple and effective discharge technique was required. A number of pre-discharge and discharge solutions were utilized to determine the best combination, based on literature findings for cnidocysts isolated from other species. The next step was to examine the puncture mechanics of the thread, by assessing its ability to puncture materials with elastic modulus ranging from 0.02 to 90,000 MPa. The puncture tests were accomplished using excised tentacles from *Physalia *as they afforded the most mechanically stable platform from which to trigger discharge. To date, the puncture mechanics of the thread have been only theoretically calculated using mass and velocity information [13]. Finally, preliminary work was conducted exploring lectins as a means to immobilize and possibly orient cnidocysts in the patch. Lectins bind strongly to sugar moieties on the surface of the cnidocyst capsule and have been used in cell culture as an attachment substrate.

## Results

### Lectin-binding examination

**Figure 2:**
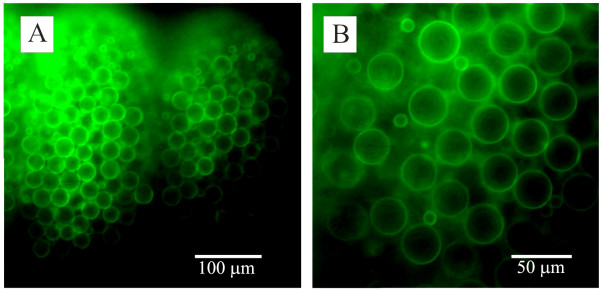
**Fluorophore-conjugated lectin binding to the cnidocyst**. Fluorescence micrographs of *Physalia *tentacles stained with FITC-conjugated lectins. (A). Lower power image of part of a tentacle stained with FITC-conjugated isolectin B4 lectin. The two clusters of stained cysts represent two cnidosacs. (B). A higher power image of cysts stained with soybean agglutinin. Two size classes of cysts are evident in this image.

A variety of fluorophore-conjugated lectins bound to the surface of the capsule (figure 2, table 1), supporting the idea that they could be used for cnidocyst immobilization. In all positive cases, binding was not localized to a particular region of the capsule, but rather spread uniformly over the entire surface of the cyst at pH 7.0 and with the ionic conditions utilized. Preliminary work using Con-A to immobilize cysts was unsuccessful. Con-A was chosen due to availability and protocols in the literature for adhering cells [14]. The adhesion by way of Con-A was not sufficient to prevent the cnidocyst rotating to absorb the mechanical force its thread encountered as it tried to penetrate something as soft as gelatin. Other lectins were not examined.

**Table 1:** Summary of fluorophore-conjugated lectin-binding to cnidocyst capsules

**Abbreviation**	**Lectin name**	**Target**	**Cyst binding**
Con-A	Concanavalin A	Glu, Man	Yes
DBA	Dolichos Biflorus Agglutinin	GalNAc	No
DSL	Datura Stramonium Lectin	GlcNAc	Yes
ECL	Erythrina Cristagalli Lectin	Gal	No
GSL-I	GSL-1 isolectin B_4_	Gal, GalNAx	Yes
GSL-II	Griffonia Simplicifolia Lectin II	GlcNAc	No
Jacalin	Jacalin	Gal	Yes
LCA	Lens Culinaris Agglutinin	Glu, Man	No
LEL	Lycopersicon Esculentum Lectin	GlcNAc	Yes
^1^PNA	Peanut Agglutinin	Gal	No
Pha-E	Phaseolus Vulgaris Erythroagglutinin	Complex N-glycans	No
Pha-L	Phaseolus Vulgaris Leucoagglutinin	Complex N-glycans	No
PSA	Pisum Sativum Agglutinin	Glu, Man	Yes
RCA_120_	Ricinus Communis Agglutinin 1	Gal, GalNAc	No
SBA	Soybean Agglutinin	Gal, GalNAc	Yes
STL	Solanum Tuberosum Lectin	GlcNAc	Yes
S-WGA	Succinulated Wheat Germ Agglutinin	GluNAc	No
UEA I	Ulex Europaeus Agglutinin I	Fuc	No
VVA	Vicia Villosa Lectin	GalNAc	No
WGA	Wheat Germ Agglutinin	GlcNAc	Yes

A variety of fluorophore-conjugated lectins were used to investigate which ones could be potentially used for cnidocyst immobilization.

Gal - galactose; Glu - glucose; Man - mannose; Fuc - fucose; GalNAc - N-acetyl galactosamine;

GlcNAc - N-acetyl glucosamine.

^1 ^stained apical end of cnidocyte in intact tentacles, but not cyst.

### Stimulation of cnidocyst discharge

Cnidocysts isolated from *Physalia *proved to be capable of discharge. Discharge was accomplished only by first drying the cnidocysts in 0.025 M to 1 M EDTA solution. In their properly dried form, any water-based solution could be used to stimulate discharge (table 2), as indicated by plus signs, denoting that over 95% of the isolated cnidocyst sample group had discharged. Note that "starting medium" in table 2 indicates both a wet discharge medium, or as the solution in which cnidocysts were dried. Alcohol-based solutions, like methanol, did not induce discharge, as denoted by a minus sign indicating less than 1% discharge. EDTA at concentrations below 0.025 M coupled with rehydration yielded very few discharged cnidocysts upon resuspension, proportional to the concentration of EDTA used for drying. Note that all chemicals were tried with at least two different concentrations to ensure test solutions were not too dilute or potent, varying between 0.1 M to 1 M, and based off successful concentrations used in the literature. Cnidocyst discharge was essentially "all-or-none" with respect to the chemicals tested, with no need for a partial discharge categorization. Unfortunately, the discharged isolated cnidocyst threads failed to puncture through the softest material evaluated (gelatin) due to capsule rotation upon thread contact with the test sample.

**Table 2:** Summary of the isolated cnidocyst discharge study

		Discharging Solution (0.1 - 1 M)*							
		
		**DI Water**	**EGTA**	**EDTA**	**Alcohols**	**KCl**	**NaSCN**	**Boric Acid**	**HCl**
**Starting Medium**	**Sea Water**	-	-	-	-	-	-	-	-
	**DI Water**	-	-	-	-	-	-	-	-
	**Spring Water**	-	-	-	-	-	-	-	-
	**EGTA**	-	-	-	-	-	-	-	-
	**EDTA**	+	+	+	-	+	+	+	+
	**Alcohols**	-	-	-	-	-	-	-	-
	**CaCl**	-	-	-	-	-	-	-	-

Isolated *Physalia *cnidocyst discharge tests, using concentrations between 0.1 M and 1 M of the indicated solutions (note that EGTA was also used at 5 mM as described in the literature[34]). Starting medium indicates both the wet medium and drying solutions before stimulation with test discharging solutions. A positive sign denotes that more than 95% of the cnidocysts discharged. A negative sign indicates less than 5% cnidocyst discharge. Discharge was only achieved when the cnidocysts were dried in EDTA and rehydrated with an aqueous solution, except in the case of DTT-induced discharge. Note that the grouping "alcohols" include ethanol, methanol, and methylated spirits (a mixture of ethanol and methanol). Experiments were conducted on at least three different batches of cnidocysts at a density of approximately 10^3 ^cysts per 50 μl.

* Note: EGTA was also used at 5 mM[34]

Interestingly, discharge was observed only when the cnidocytes were exposed fairly quickly to water. In some experiments, water was added to microwells containing the dried cnidocysts and allowed to slowly spread across the surface of the well. In such cases, the cnidocysts would not discharge, suggesting that a sudden addition of water and/or a forceful change in surface tension is required for discharge. This sudden change is not present in the slow spreading of the water droplet. Primarily water was utilized to examine this fast wetting discharge mechanism, but no gross differences were observed between water and liquids of different surface energy (i.e. EGTA). The wetting experiments were conducted in triplicate with each chemical. Determining the specific mechanism of this wetting speed phenomenon requires more research.

Addition of 0.5 M DTT solution prepared in methanol yielded very slow discharge of the threads when the cnidocysts were buffered (wet) or dried in EDTA, which was quite unlike the very fast discharge when exposed to water. Notably, DTT was the only solution that yielded discharge of the cnidocyst thread in a "wet" state, suggesting a separate discharging mechanism than with aqueous solutions. The slow uncoiling of the thread delayed about 1 min-post stimulation and required another minute for complete uncoiling. In the dried cnidocysts, the threads only partially everted. Contrastingly, the cnidocysts buffered in EDTA solution (wet) completely discharged the thread. The unraveling occurred at a slightly faster rate and resulted in complete separation of the thread from the capsule.

Electric field stimulation of cnidocyst discharge proved to be unsuccessful in all cases; no eversion of the thread was observed. Notably, the intact cnidocysts moved when exposed to an electric field, with a majority migrating towards the anode, suggesting a net positive charge.

### Mechanical puncture tests

The tentacle-contained *Physalia *cnidocysts were capable of puncturing PDMS and all materials tested with an elastic modulus below 1 MPa as shown in table 3. However, no thread penetration was observed through nitrile or materials harder than 2 MPa. This suggests that the cnidocyst thread for this animal has an elastic modulus puncture threshold of about 1-2 MPa. In all positive penetration cases, more than 10 threads punctured the material in each trial, making penetration classification unambiguous. The penetration success rate for all positive cases was 100%.

**Table 3:** Summary of tentacle-contained cnidocyst penetration of various materials

**Material**	**Elastic Modulus (MPa)**	**Penetration**
Gelatin	0.02	+
Polyacrylamide	0.06	+
Teflon	0.10	+
Latex	0.80	+
PDMS	1.00	+
Nitrile	2.60	-
Polyvinylchloride	250	-
Polycarbonate	2,000	-
Aluminum	70,000	-
Glass	90,000	-

List of test materials and their respective elastic moduli. The ability of tentacle-contained cnidocysts from *Physalia *to puncture these materials is also listed. Note that the elastic modulus of human skin is approximately 0.13 MPa [35]. A positive sign denotes successful penetration of at least some of the threads in all three trials. A negative sign indicates a lack of successful penetration in all trials. In positive cases, thread penetration occurred in all three trials.

## Discussion

**Figure 3:**
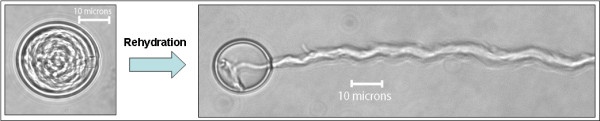
**Images depicting before and after cnidocyst discharge via rehydration with water**. Isolated *Physalia *cnidocyst dried in EDTA (left). The capsule remains spherical when dried in this solution. The cnidocyst rapidly everts its stinging thread following rehydration with distilled water (right). Note the ~1 mm thread continues beyond the area depicted in the picture.

**Figure 4:**
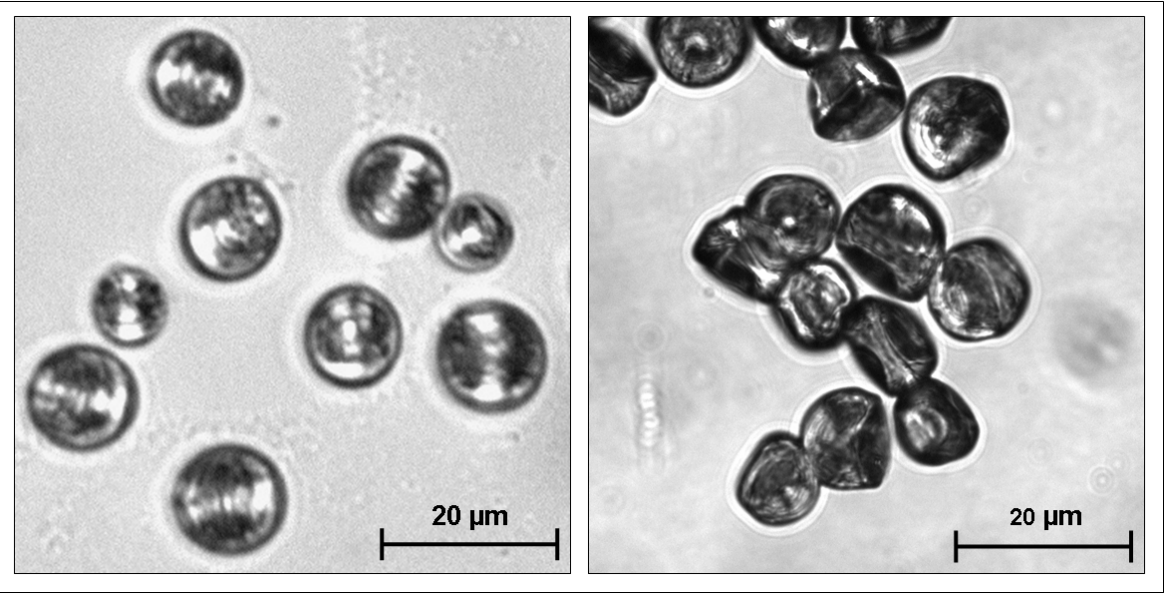
**Comparison of cnidocysts dried in EDTA vs. any other aqueous solution**. Brightfield images comparing cnidocysts dried in EDTA (left) vs. any of the other aqueous drying solutions tested (right). Cnidocysts discharged when in the "pseudo-hydrated" form depicted on the left.

A means of isolated *Physalia *cnidocyst discharge in a stable, dried form has been discovered. Upon drying cnidocysts in EDTA solution, the cnidocyst could be stimulated to discharge in any aqueous solution including distilled water (Figure 3). No other drying solution yielded the same result, including the more potent calcium chelator EGTA. The exact mechanism of discharge in this way is unknown, but could be explained by the difference in morphology between EDTA-drying and drying in other solutions. When dried in EDTA, the cnidocysts possess a pseudo-hydrated morphology where intracapsular osmotic pressure is still maintained (Figure 4). This is in stark contrast to the appearance of cnidocyst dried in any other solution. In the latter case, the cnidocysts shrivel when dried. Upon addition of an aqueous solution, a majority of the EDTA-dried cnidocyst discharge their threads. However, the shriveled cnidocysts dried in other solutions simply rehydrate upon exposure to water, but do not discharge. The reason for this incomplete drying of the cnidocysts in EDTA solution could be explained by blockage of pores on the surface of the capsule. EDTA is a chelator for a number of cations besides calcium, including magnesium and other metal ions. Perhaps these cations play an important role in ion translocation across the surface of the capsule, which is highly permeable to a number of charged atoms [9]. Due to the maintenance of the osmotic pressure within the capsule in the pseudo-hydrated form, the thread is allowed to rapidly evert via the osmotic pressure mechanism.

Unfortunately, isolated cnidocysts were unable to penetrate even gelatin, probably because the unrestrained capsule was rotating to relieve the pressure induced when the thread encountered the test material. Lectins were considered as a means to immobilize the capsule and prevent this reorientation, but did not yield thread penetration even in gelatin. This penetration failure could be attributed to the fact that the substrate-lectin bond, while physically strong, was not sufficient to counter the large moment generated by the rotational forces created by the capsule reorienting. Liu *et al *describe a procedure where plant lectins (E-PHA) can be covalently bound to a glass coverslip by compression and photo-crosslinking [15]. A similar procedure could be used to bind Con-A or STL to a glass substrate, ensuring a stronger bond. Another possibility for the lack of immobilization is that drying the cnidocysts unbinds the cnidocysts from the Con-A-coated substrate. Water molecules have been shown to play an important role in the binding kinetics of a number of lectins [16-18]. The cnidocyst discharge event could occur before the water component of the discharging solution can restore the lectin bond. Finally, the lectins could be degraded in a dried form, thus cleaving the capsule attachment to the substrate. More work is required to fully assess the applicability of lectins for capsule immobilization. Another possibility that could be explored is whether there are differences in discharge mechanisms and forces between the rehydration method of isolated cnidocysts and physiological discharge in an intact animal.

DTT was used to investigate the importance of disulfide bonds in cnidocyst thread discharge. DTT prepared in methanol yielded a slow discharge of the cnidocyst thread, with the speed dependent on the hydration state before addition of DTT. The slow uncoiling in both cases could be explained by the fact that disulfide bonds may be important in maintaining the tightly coiled structure of the cnidocyst thread [19], in addition to maintaining the capsule wall integrity reported elsewhere [12,20]. As the DTT slowly diffuses into the capsule and cleaves these bonds, the tension associated with the thread coil overwhelms the remaining bonds in the capsule wall and thread itself, as visualized by this slow uncoiling. The lack of fast and full discharge could be due to the lack of extreme pressure or electrostatic repulsion forcing the thread. Thus, DTT-induced discharge may be permitting observation of the thread tension component of discharge. Surprisingly, DTT did not cause discharge in cnidocysts buffered or dried in any of the other chemicals tested, suggesting that the cleavage of disulfide bonds is not the only mechanism and the EDTA plays an important role beyond that of maintaining osmotic pressure. The mechanism is not purely calcium dependent, as cnidocysts buffered or dried in EGTA solution did not fire, so perhaps the affinity of EDTA for other cations plays a role in permitting thread eversion.

Fast-occurring biological phenomenon due to structure tension and calcium translocation is not a new concept in biological systems. Shin *et al *quantified the force of a biological spring in the ability of a *Limulus polyphemus *sperm actin bundle to puncture the egg envelope, without the presence of a build-up of hydrostatic pressure as in the case of a cnidocyst [21]. The mechanism for bundle extension involves calcium disassociation from the actin-crosslinker scruin, which then permits the initially overtwisted actin bundle to rapidly untwist and therefore extend [22]. Sperm bundle pressures exceeded 1.6 MPa, suggesting that the spring-like configuration of the cnidocyst thread could play a small role in the 7 GPa thread tip pressure found in *Hydra *[13]. The contraction dynamics of *Vorticella convallaria *are also very relevant to the present study. The protozoan attaches to a substrate via a stalk and rapidly contracts when stimulated. The organism has shown to change nearly 5000 times its length in less than a second. As with cnidocysts and horseshoe crab sperm, high intracellular calcium levels are found within the cell and mediate the explosive process [23]. When calcium is bound to the spasmoneme enclosing the stalk, the repulsive forces generated by the negatively-charged spasmin backbone are neutralized, yielding contraction [24]. In the calcium unbound state, the spasmin protein chains repel one another, resulting in stalk extension. This process is somewhat similar to the proton gradient theory applied to cnidocysts in that charge repulsion and relocation of ions causes an explosive biological process [10]. A subsequent study on *Vorticella *quantified speed through viscous media to elucidate the contraction dynamics [25]. Stalk contraction was demonstrated to occur at a faster rate than the velocity of the cell body, suggesting that the rate of contraction is ultimately dependent on the power delivered by the spasmoneme, as well as the rate at which calcium can be released from the stalk [22].

Cnidocyst discharge by chemical means is not the only method previously reported and may not even be the most efficient. Electrical discharge has worked for a number of groups with a wide variety of cnidarian species [1,5,26]. Following protocols used for stimulating discharge of the freshwater cnidarian *Hydra *[13], electrical stimulation of cnidocyst eversion was attempted on isolated *Physalia *cnidocysts, but proved unsuccessful. The observation that many of the cnidocysts migrated towards the anode suggests a net positive charge of the cnidocysts, which corroborates with other observations in the literature, where researchers discovered that the stinging capsule operculum is positively charged while the basal end of the cnidocyst is neutral. The positive charge finding prompted the use of dielectrophoresis patterning of the cnidocysts as part of a possible patch fabrication method [27,28]. The results were ultimately not consistent or effective.

In the present study, the puncture mechanics of the *Physalia *cnidocyst thread have been characterized for the first time. As expected, the threads were capable of puncturing relatively hard materials (PDMS = 1 MPa) compared to human skin (0.13 MPa). This information will be useful in designing the patch. A material used for the membrane separating the cnidocyst within the well and the patient's skin should be made of a material with an elastic modulus of less than 1 MPa, such as PDMS or something softer.

The binding of lectins to the surface of the cnidocyst capsule is attractive as a possible means of immobilizing the cnidocysts within the containment wells of the patch. The cnidocysts could be bound to the separation membrane or bottom of the well. Some of the cnidocysts would fire into through the membrane and into the patient's skin. A binding molecule that exhibits heterogeneous binding to the capsule wall is ultimately preferred which would permit pre-orientation of the cnidocysts in addition to immobilization and ensure that a majority of the cnidocysts would discharge in the correct direction. Only homogeneous binding was observed using the lectins tested with *Physalia*. It should be noted that no attempt was made to confirm that the observed binding patterns of different lectins mirrored the distribution of the relevant sugar moieties since we were not interested in the biochemistry of the cells, per se, merely whether or not one might be able to capitalize on structural heterogeneity to orient immobilized cysts. Lotan et al suggested the use of other bioadhesives that could be used, including biological glues (e.g. BIOBOND™), bacterial fimbrins, and polylysine meshes, among many other means [29]. The charge heterogeneity of the cnidocyst capsule could be utilized as well, such as by binding the operculum to a negatively-charged material that would serve as the puncture membrane of the patch.

The rest of the patch could be made of a harder material, such as silicon. This would prevent those cnidocysts that were not oriented from discharging in the wrong direction. Silicon is a hard material that permits fabrication of complex designs. A microfluidic network could be incorporated into the silicon component of the patch. This network could deliver water to the individual containment wells, inducing discharge of the cnidocysts. Additionally, multiple drugs could be independently delivered using the same patch by localized stimulation of cnidocysts with the microfluidic network. Ultimately, it is hoped that the cnidocysts could be genetically re-engineered to not only deliver but also manufacture the desired drugs for injection. Creation of an interstitial cell line for cnidocytes would greatly aid in the re-engineering process but this would only be applicable to drugs that can be produced biologically.

## Conclusion

The ability to utilize cnidocysts as a functional material is attractive based on their robust mechanism for thread eversion. Tentacles of *Physalia *were able to penetrate PDMS, which is a welcomed result due to the ease of fabrication. A protocol for discharging isolated cnidocysts was also identified; however they were not able to penetrate any material tested due to the inability to anchor them to a substrate. To explore means of immobilizing and possibly orienting the cnidocysts prior to firing, fluorophore-conjugated lectin binding to the capsule wall was visualized. Several lectins bound homogenously to the surface of the *Physalia *capsules, suggesting the possibility for cnidocyst immobilization but not orientation. The information determined from this study will be useful in applying cnidocyst to drug delivery applications.

## Methods

### Isolation of cnidocysts from Physalia

*Physalia physalis *specimens (multiple animals) were collected in shallow water off beaches near the University of Florida Whitney Laboratory for Marine Bioscience, Marineland, Florida. Specimens were typically about 10 - 15 cm in float length. Excised, full length tentacles from *Physalia *were soaked in distilled water at 4°C for one week, with multiple rinses. Because of their density, isolated cysts readily accumulated at the base of the vessel allowing the supernatant to be removed and the slurry of cysts to be collected. The slurry was washed three times, stored in distilled water at 4°C, and used within 6 months of isolation.

### Stimulation of isolated cnidocyst discharge

Numerous combinations of pre-discharge incubation and discharge-inducing solutions were tested as a way of inducing discharge of isolated cnidocysts (table 2). The chemicals were used at various concentrations based on reports from the literature demonstrating discharge of cnidocysts in other species [30,26,34]. The stock cnidocyst solution used contained a high concentration of cnidocysts suspended in distilled water. To test the various "wet" and "dry" discharge solutions, the stock solution was mixed 1:10 with the test solution of choice for rinsing purposes. This mixture was then centrifuged at 700 RCF to pellet the cnidocysts. The supernatant rinsing solution was removed and the test solution was then added to the original cnidocyst pellet. Once resuspended, the cnidocyst solution was pipetted into microwells of a custom-fabricated polydimethylsiloxane (PDMS) well-plate for high-throughput testing. The well plate was essentially a slab of PDMS containing 5 mm diameter holes and was oxygen plasma-bonded to a glass slide. For "wet" discharge studies, the cnidocysts were allowed to incubate in the test solution for 1 h and then stimulated to discharge chemically. In the "dry" studies, the cnidocysts were placed in a dehumidified room at 45°C to rapidly dry the organelles. Once dried, the test solutions were pipetted atop the cnidocysts to induce discharge.

Of note, pure methanol was used as a control for investigating dithiothreitol-induced discharge. Dithiothreitol (DTT) cleaves disulfide bonds and is a common reducing agent in molecular biology. As with the rest of the chemicals examined, it was utilized on both wet and dry cnidocysts.

Electric current-mediated discharge was also attempted on isolated *Physalia *cnidocysts. The recent paper suggesting a proton gradient and electrostatic repulsion mechanism of discharge encouraged the use of electric fields to stimulate the cnidocysts [10]. A range of frequencies (0 Hz to 16 MHz) and amplitudes (10 mV to 30 V) were used to induce discharge of cnidocysts positioned atop electrodes in both DI water and conductive solutions. For the pilot experiments, a cnidocyst suspension in sea water was pipetted between two parallel exposed wires in a setup similar to Holstein and Tardent [13]. Multiple wire separations of between 1 mm and 10 mm were used. A variety of other solutions (see table 2) were tested to examine the effects of the medium on discharge. To improve the reproducibility and handling of the experiment, a circuit board was designed using metal-etching coupled with conventional photolithography. Both 1 mm and 10 mm spacing were used in designing the photolithography mask. Each stimulation method (both chemical and electrical) was applied to large batches of cnidocysts (>10^3^) over 3 trials (N = 3) to ensure reproducibility.

### Preparation of excised tentacles

Small sections of *Physalia *tentacles (2 cm) were excised from a single *Physalia *specimen's tentacles maintained in an aquarium at the Whitney Laboratory for Marine Bioscience (Marineland, FL). These were then anaesthetized by incubation in isotonic (0.37 M) MgCl_2 _mixed 1:1 with sea water. Once anaesthetized, the tentacles were further sectioned into 5 mm pieces. These were then used for the mechanical puncture tests.

### Tentacle-contained cnidocysts puncture tests

**Figure 5:**
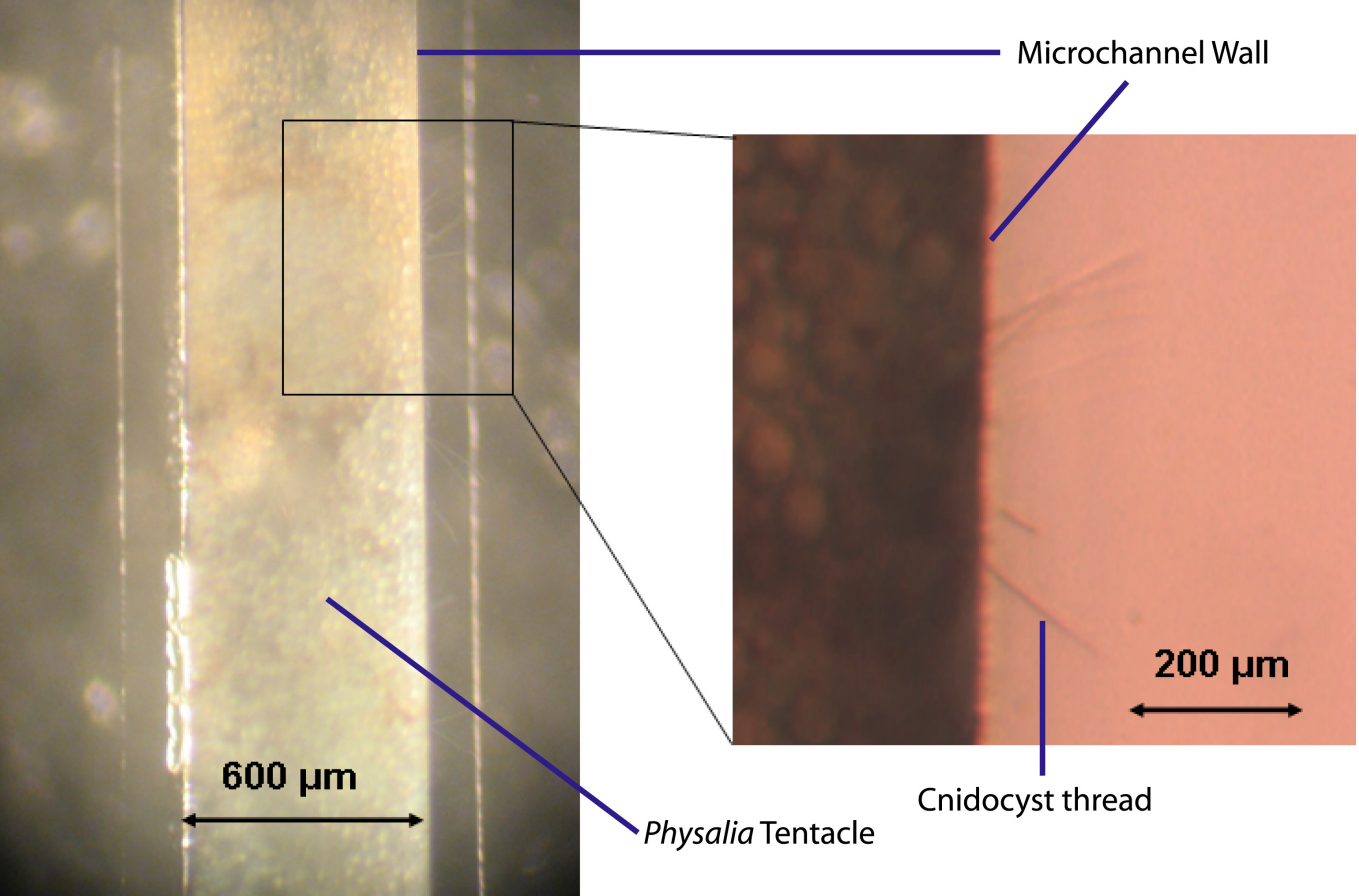
**Thread penetration into the PDMS microchannel wall**. Brightfield macroscope (left) and microscope (right) images depicting thread penetration into the PDMS in a custom-made microchannel.

Excised tentacles from *Physalia *were used to characterize the ability of the cnidocysts to puncture a variety of materials with a range of elastic moduli (table 3). The materials ranged from 0.02 MPa (glutaraldehyde-crosslinked gelatin) to 90,000 MPa (glass). The silicone elastomer polydimethylsiloxane (PDMS) was used as a starting point. For reference, human skin ranges from about 0.1-0.15 MPa [35]. PDMS microchannels (600 μm wide x 300 μm tall) were fabricated using conventional photolithography and replica molding techniques. The *ex vivo *tentacles were then pulled within the channels. Discharge stimulation was induced by addition of the potent calcium chelator ethylene glycol tetraacetic acid (EGTA) solution, since calcium ion translocation plays a critical role in discharge [9]. EGTA yielded the most discharged threads for *Physalia *and has been used with great effectiveness for discharge stimulation of other cnidarian species as well, including isolated capsules [34]. Thread penetration into the surrounding PDMS walls was easily observed with both a dissecting and compound microscope (Figure 5).

For testing with all other materials, the excised tentacle was placed atop a glass slide, and then a 200 μm thick film of each material (McMaster-Carr, Chicago, IL) was placed on top of the tentacle. Discharge stimulus occurred via mechanical stimulation of the cnidocil by tweezer probing, which was more efficient than EGTA stimulation. Thread penetration up through the material was visualized using a dissection microscope. Each material was tested at least 3 times. To be denoted "positive" penetration, one or more threads must have penetrated the material in all three trials.

### Lectin-binding

Tentacles were excised from freshly collected *Physalia *and anaesthetized in a 1:1 solution of isotonic MgCl_2 _and natural, unfiltered sea water for 30 min. They were then lightly stretched and pinned to a layer of PDMS using cactus spines before being fixed in 4% phosphate-buffered paraformaldehyde for 2 h at room temperature. Following four, 30 min rinses in phosphate-buffered saline containing 0.25% Triton-X 100 (PBS-T) followed by two additional 30 min rinses in PBS-T containing 1% goat serum, tentacle fragments were incubated overnight at 4°C in 1:1000 dilutions of a range of FITC-conjugated lectins (Vector Labs) as listed in table 1 at pH 7.0. Following five, 30 min rinses in PBS-T, the tentacles were mounted on a microscope slide in 90% glycerol containing the anti-fading agent paraphenylenediamine (Sigma). The protocol for staining of isolated cysts was the same except that isolated cysts were fixed on gelatin-coated microscope slides for 1 h. Rinsing protocols were the same as for whole tentacles, but lectin incubation was reduced to 1 h at room temperature. Stained tissues were examined using either a Leitz DMRD fluorescence microscope (Wetzlar, Germany) or a Leica SP2 Confocal (Bannockburn, IL).

Lectins were also evaluated as a means to immobilize the cnidocysts, preventing capsule rotation during discharge. Lectins were adhered to a glass slide using a protocol for neuron culture vessel coating [14]. The cnidocysts suspended in ethylenediaminetetraacetic (EDTA) solution were allowed to dry and then a wet piece of gelatin was placed on top to stimulate discharge.

## Competing interests

The authors declare that they have no competing interests.

## Authors' contributions

SO carried out the experiments and drafted the manuscript. PA provided materials for the experiments. DE conceived of the study, and participated in its design and coordination. All authors read and approved the final manuscript.
